# Supplementary material for: Complex Diagnostics of Non-Specific Intellectual Developmental Disorder
Source: Int J Mol Sci. 2022 Jul 14;23(14):7764. doi: 10.3390/ijms23147764 (PMC9323143; doi:10.3390/ijms23147764)
Supplement: Supplementary file 1 [file ijms-23-07764-s001.zip › Supplementary Matherial S2.pdf]

Previously undescribed genetic variants, a brief description of the phenotype.

Patient D171. *SMARCA2* NM_003070.5:c.1258C>T; NP_003061.3:p.(Arg420Cys)

Pathogenic variants in this gene are often described in NIDD with hypotonia and behavioral deviations [1]; they also occur in Nicolaides-Baraitser [2] and Coffin-Siris syndromes [3]. According to HGMD, missense substitutions located closer to the N-terminal region of the protein are more often described in NIDD, while substitutions at the C-terminus are more often described in Coffin-Siris and Nicolaides-Bareitser syndromes. There appears to be no dependency of the clinical features on the location of the variants within or outside the functional domain. Our proband was an 11-year-old boy, the identified variant the closest to the N-terminus among all those described, which suggests that it should lead to a milder clinical picture without pronounced dysmorphias, which we nevertheless observed in the patient’s phenotype: triangular face, down-slanting palpebral fissures, everted nostrils. The patient's speech consisted of syllables, there are also signs of autism and dysarthria. The variant is located in the HAS domain and can disrupt the binding of the putative global transcription activator SNF2L2 (*SMARCA2*) to DNA.

Patient D177. *ACTL6B* NM_016188.5:c.554T>C; NP_057272.1:p.(Leu185Pro)

Variants in the *ACTL6B* gene can lead to pathology in both in the heterozygous and in the homozygous state. At the same time, according to the literature, the development of recessive forms of NIDD is mainly caused by LoF variants (stop-gain, frameshift variants), while dominant forms are caused by gain-of-function variants (mainly missense) [4] (Figure S1). Most patients with *ACTL6B*-associated ID are characterized by severe developmental delay, epileptic encephalopathy, and spasticity [4,5], however, patients with autism and NIDD alone have also described [6]. One family that we studied included 3 siblings with a clinical picture of IDD of varying severity. Of the dysmorphic features, only hypotelorism was observed in all three siblings; the 3-year-old boy showed epileptic activity on the EEG, but no seizures were registered, the middle 14 years old sister had febrile seizures once, and the older brother who suffered from progressive epilepsy for 10 years died at the age of 17 from a fall. Exome sequencing revealed a homozygous p.Leu185Pro variant in the proband, while the same variant was detected in the homozygous state in siblings by Sanger sequencing.

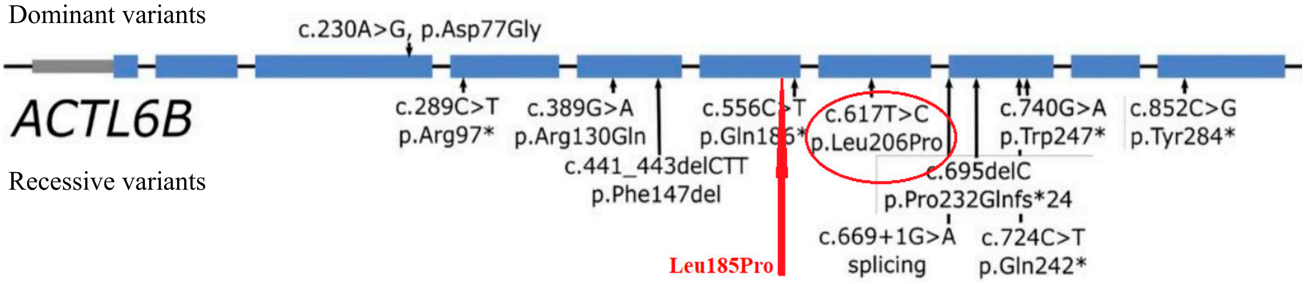

Figure S1. Distribution of variants in the *ACTL6B* gene, exons 1-10.

Though missense variants are known to cause dominant forms of the disease, there is a p.Leu206Pro variant (Figure S1), which is located close to the variant identified in this work and shown to have the same effect on differentiating neuronal cells as the LoF variants [4], that cause the recessive form. The *ACTL6B* gene encodes a tissue-specific subunit of the BAF complex, BAF53b, which is required for postmitotic neural development [7]. In view of this, *ACTL6B*-associated IDD can also be classified as a BAFopathy.

Patient D198. *RAI1* NM_030665.4:c.629C>G; NP_109590.3:p.(Pro210Arg)

This gene has been known since the 20th century as the cause of the frequent Smith-Magenis syndrome [8]. In most cases, this syndrome is caused by a ~4Mb deletion at the 17p11.2 locus. However, 5-10% of cases are due to SNV in the *RAI1* and often have a milder phenotype [9]. In addition, missense variants are described in NIDD [10] and autism [11]. In this study, a *de novo* missense variant p.Pro210Arg was detected in a 14-year-old girl; the clinical picture did not contradict the Smith-Magenis syndrome, but was insufficient to establish this diagnosis clinically. The patient was characterized by obesity, epicanthus, hypoplasia of the middle third of the face, long filter, wide hands and feet, autoaggression, and decreased pain sensitivity. In contrast to patients with the classical form of the syndrome, the patient had normal height; neither hypotension nor ophthalmic and otolaryngological abnormalities were detected; a small number of minor developmental anomalies that were not very pronounced were observed. No functional domains have been identified in this region, but according to the Uniprot data, region 1-261 is predicted to be "Disordered" and may contain parts that are responsible for interaction with other proteins.

Patient D298. *GRIN1* NM_007327.4:c.1918G>C; NP_015566.1:p.(Ala640Pro)

GRIN1-associated pathology is the closest in its clinical manifestations to isolated ID. Phenotypic features are variable and often absent, with hypotension as the most common feature; sometimes seizures and hyperpyretic disorders are described [12]. The disease can be inherited by both AD and AR types, although AR forms have a more severe course with a generally similar clinical picture, which is due to a dominant-negative effect [13]. A *de novo* variant p.Ala640Pro was detected in an 18-year-old girl with NIDD and microcephaly; no facial abnormalities and no epileptic activity on the EEG were observed. This variant is located in the transmembrane helix and can enhance the function of transmembrane calcium ion import into the cytosol [13], which leads to the gain-of-function effect and the dominant form of the disease, respectively.

Patient D332. *NEXMIF* NM_001008537.3:c.2667G>A; NP_001008537.1:p.(Trp889Ter)

Pathogenic variants in the *NEXMIF* lead to X-linked IDD, type 98. The disease is often accompanied by myoclonic atonic epilepsy, which is superimposed on eyelid myoclonus; some patients had IDD without epilepsy, which was more severe in males. All described variants are LoF [14]. The variant p.Trp889Ter was identified in a 2-year-old boy. The identified stop codon is

located further than most of the previously described pathogenic variants (ranging from 146 to 856 codons), which may explain the milder phenotype – the boy had only a laterally compressed skull. The variant p.(Trp889Ter) disrupts the neurite extension and migration factor that is involved in neurite outgrowth by regulating intercellular adhesion via the N-cadherin signaling pathway [15].

Patient D336. *TRIP12* NM_004238.3:c.3759_3760del; NP_004229.1:p.(Gly1254IlefsTer36)

This gene is associated with autosomal dominant IDD, type 49, accompanied by nonspecific facial dysmorphism and in some cases autism [16]. The severity of the clinical picture does not depend on the type of variant identified [17]. A 4-year-old girl with mild motor developmental delay and mild facial dysmorphism (macrostomia, long filter, adhering lobes), without epileptic activity on the EEG and seizures, had a frame-shift variant p.Arg1253fs., leading to the loss of function of E3 ubiquitin-protein ligase (*TRIP12*) involved in the ubiquitin fusion degradation pathway and the regulation of DNA repair.

Patient D364. *PGAP3* NM_033419.5:c.827C>T; NP_219487.3:p.(Pro276Leu)

The disease caused by pathogenic variants in this gene is one of six in the phenotypic series of hyperphosphatasia and IDD. The severity of clinical manifestations of the disease are varies from severe syndromic forms with multiple organ defects to NIDD [18]. *PGAP3*-associated IDD is an autosomal recessive disease characterized by psychomotor retardation and postnatal microcephaly [19]. The patients in this study were 10- and 9-years-old sisters with severe developmental delay, thickened nose bridge, joint hypermobility and normal head size. The examination revealed a homozygous missense variant p.Pro276Leu in both girls. The level of alkaline phosphatase was increased to 809U/L (normal level: 129-417) and 1059U/L respectively, which functionally reflects a disruption of the glycosylphosphatidylinsitol pathway.

Patient D375. *DYRK1A* NM_001396.5:c.572_575del; NP_001387.2:p.(Lys191ThrfsTer6)

Autosomal dominant IDD, type 7, accompanied by microcephaly, nonspecific facial dysmorphias and MRI changes, as well as autistic behavioral traits and epilepsy [20]. The patient, a 13-year-old boy, had motor developmental delay, epilepsy, microcephaly, wide interdental spaces, protruding incisors, lower micrognathia, long eyelashes with irregular growth, divergent strabismus, high-backed nose, obesity, thin long fingers, and flat feet. A de novo frameshift variant p.Lys191fs was found in the *DYRK1A*. This variant has been previously described in a patient with autism, [21] and has been shown to result in the loss of function of dual specificity tyrosine-phosphorylation-regulated kinase 1A.

Patient D473. *ADNP* NM_015339.5:c.2155del; NP_056154.1:p.(Tyr719ThrfsTer9)

Pathogenic variants in the *ADNP* have been described in the Helsmoortel-van der Aa syndrome; the protein product of this gene interacts with the BAF complex, however, the

phenotypic manifestations of the syndrome are polymorphic and often nonspecific. The only important distinguishing feature of this syndrome is early teething [22]. In recent years, however, mutations in the *ADNP* gene have also been shown to be one of the most common causes of IDD with autism [23]. In this study, we identified a frameshift variant p.Tyr719ThrfsTer9 in a 4-year-old boy with intellectual disability, tower-shaped skull, protruding forehead, flat occiput, highlighted eyebrows, tented upper lip, clinodactyly of the 5th fingers, valgus legs and feet, and micropenis.

Patient D473. *BRD4* NC_000019.9:g.15349980_15349986dup;
NP_490597.1:p.(Glu1225GlnfsTer16)

In the literature, 5 patients with pathogenic variants in the *BRD4* have been described [24,25]. The facial phenotype of some patients is similar to Cornelia-de Lange syndrome (microcephaly, synophrys, long eyelashes, depressed nasal bridge, uptilted nasal tip, and low-set ears) [25]. Genetic and phenotypic polymorphism is not well understood. The patient in this study was a 29-year-old man with poor posture, narrow palpebral fissures, small hands and feet. Exome sequencing revealed a frameshift variant p.Glu1225fs. This variant is located in exon 18 (out of 20), that is, much closer to the end of the protein then the previously described variants in exons 4 and 6, which may explain the different phenotype compared to the previously described patients who had a disrupted NET domain which mediates interaction with a number of chromatin proteins involved in the regulation of transcription.

Patients D543 and D601. *SCN2A* NM_021007.3:c.1499_1500del;
NP_066287.2:p.(Glu500AlafsTer21) and NM_021007.3:c.2380G>A;
NP_066287.2:p.(Gly794Arg)

Pathogenic variants in this gene can cause several distinct conditions such as autism [26], early epileptic encephalopathy [27] and NIDD [28] grouped into developmental epileptic encephalopathy and type 11 (MIM: 613721). In addition, episodic ataxia and familial benign infantile seizures have been described. In total, more than 400 different pathogenic variants in the gene have been described. The LoF variants are more common in IDD, while missense substitutions are more common in early epileptic encephalopathy [29]. We identified two variants in this gene in two unrelated patients. A 5-year-old boy with delayed motor development, hypotension and obesity had a *de novo* frameshift variant p.Lys499fs. Another 5-years-old boy without any abnormalities, except IDD, had a *de novo* missense variant p.Gly794Arg was detected, located in one of four internal repeats (with 5 hydrophobic segments and 1 positively charged segment). It can lead to channel dysfunction. Both boys showed no epileptic activity on the EEG.

Patient D543. *AHDC1* NM_001029882.3:c.1181_1182del; NP_001025053.1:p.(Cys394SerfsTer122)

In 2014, F. Xia et al. described *AHDC1*-associated pathology as a syndromic speech disorder with hypotension and sleep apnea [30], after which it was named the Xia-Gibbs syndrome. Subsequently, H. Yang et al. in 2015 described the same disease as an IDD with nonspecific clinical features, since the features of dysmorphogenesis are not characteristic of all patients [31]. In our study, the patient was a 6-year-old girl with motor retardation, moderate atrophic changes and periventricular leukopathy on MRI and phenotypic features (sparse dry hair, low auricles, enophthalmos, superciliary arch hypoplasia, ulnar deviation of the 1st finger, brachydactyly of the fingers). Examination revealed a *de novo* frameshifted variant of p.Cys394fs, which leads to the loss of function of AT-hook DNA-binding motif-containing protein 1.

Patient D659. *DNMT3A* NM_175629.2:c.1443C>A; NP_783328.1:p.(Tyr481Ter)

DNMT3A-associated overgrowth syndrome or Tatton-Brown-Raman syndrome in honor of Katherine Tutton-Brown and Naznin Raman who first described it [32]. The main clinical manifestations of the syndrome are IDD and high height (80%), in addition, joint hypermobility, obesity, autism and seizures are often observed [32]. The proband was 10-year-old boy with macrocephaly and complaints of learning difficulties, progressive visual impairment, and rapid fatigue; he was found to have a *de novo* variant p.Tyr481Ter that leads to the loss of function of DNA (cytosine-5)-methyltransferase 3A. The boy's height was at the upper limit of normal, hypermobility of the joints and dysmorphic features (downslanting palpebral features, epicanthus, convergent strabismus, backward-rotated ears, thin upper lip, protruding incisors, crowded growth of teeth) were observed.

Patient D680. *POGZ* NM_015100.4:c.600dup; NP_055915.2:p.(Gly201TrpfsTer114)

Pathogenic variants in this gene were identified in a group of patients with IDD and autism, the researchers identified common phenotypic features among them and suggested that this disorder is syndromic [33]. Subsequently, *POGZ*-associated IDD was named the White-Sutton syndrome. However, the presence of these features does not make it possible to distinguish this condition from other similar conditions, therefore, it is incorrect to call it syndromic and the name has not taken root in the literature [34]. The proband, a 7.5-year-old boy with epileptic activity on the EEG and no history of seizures, had enlarged mammary glands and vellus hair on his arms. The features of the facial phenotype were not pronounced: low-set ears with massive adherent lobes, high palate, short upturned nose. Examination revealed a *de novo* heterozygous variant p.Gly201fs which leads to a frameshift and formation of a premature stop codon at position 315.

Patients D685. *SATB2* NM_015265.4:c.414_415del; NP_056080.1:p.(Val139GlyfsTer69) and D886 NM_015265.4:c.696dup; NP_056080.1:p.(Lys233Ter)

SATB2-associated syndrome (Glass syndrome) is a recently described disorder characterized by developmental delay/mental retardation with no or limited speech development, craniofacial anomalies, behavioral problems, dysmorphic features, and palate and dental anomalies [35]. In some sources, the disease is described as an IDD with cleft palate [36] or *SATB2*-associated disorder with a Rett-like phenotype [37]. We identified two variants in this gene in two unrelated patients. Patient 1 was an 11-year-old girl with motor developmental delay and epilepsy. Examination revealed microcephaly, high forehead, deformed ears, hypertelorism, long filter, high palate, oligodontia, anomaly in the location and structure of the teeth, sharp chin, pterygoid shoulder blades, dolichomorphic limbs, arachnodactyly of the lower and upper limbs. NGS identified revealed a *de novo* frameshift variant p.Thr138fs. Patient 2 was a 9-year-old boy with delayed motor development, mild periventricular leukopathy on MRI, microcephaly, facial dysmorphism (microstomia, narrow lips, high palate, multiple dental caries, dysplastic soft backward-rotated auricles). NGS identified a *de novo* stop-gain variant p.Lys233_Val234delinsTer. Both variants lead to the loss of function of DNA-binding protein SATB2.

Patient D737. *TUBB* NM_178014.4:c.623_624del; NP_821133.1:p.(Tyr208Ter)

One of the clinical manifestations of pathogenic variants in the *TUBB* gene is congenital malformations of the brain, manifested by focal polymicrogyria and microcephaly [38]. Also, pathogenic de novo variants in the region of the N-terminus of *TUBB* are associated with the "Michelin syndrome" characterized by ring-shaped skin folds on the limbs of children under the age of 3 years and IDD [39]. In the literature, only missense variants are described as the cause of the disease, however, *TUBB* has a high sensitivity to LoF variants (pLI = 0.98). In this study, the proband was a 39-year-old male patient with a history of delayed motor development, normal height and weight and an extra Y chromosome. Examination revealed kyphosis in the thoracolumbar region, strabismus, ocular hypertelorism, short filter, and protruding lower jaw. NGS identified a heterozygous *de novo* variant p.Tyr208fs. Previously undescribed features, such as thoracolumbar kyphosis, protruding mandible, and normal head circumference, may be associated with a previously undescribed type of LoF variant.

Patient D755. *ZBTB18* NM_205768.3:c.583C>T; NP_991331.1:p.(Arg195Ter)

This gene are is associated with an autosomal dominant IDD, type 22, which can be accompanied by various clinical features, including microcephaly, anomalies of the corpus callosum, and seizures [40]. The patient was a 6.5-year-old boy with microcephaly and periventricular leukomalacia on MRI. On examination, tall stature, clinodactyly of the little fingers of the hands, motor awkwardness when walking, flat-valgus feet were noted. NGS revealed a *de novo* stop-gain variant p.Arg195Ter. This variant has previously been identified in a girl with atypical Rett syndrome [41] described as NM_006352:c.C556T:p.(R186X) in *ZNF238*.

Patient D843. *FRMPD4* NM_014728.3:c.1411G>T; NP_055543.2:p.(Glu471Ter)

The 104-th type of X-linked IDD is one of the rare ones; only 10 patients from 4 unrelated families have been described. This type of IDD may be accompanied by non-specific phenotypic features; tremor, strabismus and seizures are rarely observed [42]. The proband was 16-year-old boy with mild signs of autism and subtle phenotypic features (protruding nose, thin back and wide tip of the nose, large protruding ears, hypertelorism, exophthalmos, multiple nevi, hyperelasticity of the skin). A *de novo* hemizygous variant p.Glu471Ter was identified, leading to the loss of function of FERM and PDZ domain-containing protein 4.

Patient D837. *RBFOX1*NM_145891.3:c.1252T>G; NP_665898.1:p.(Tyr418Asp)

Variants in this gene have been described in NIDD with autism and single non-specific phenotypic features such as an downslanting palpebral features, slit, and smooth filter [43]. However, there have been only three descriptions of point substitutions, mainly intragenic deletions of one or several exons or large rearrangements affecting the whole gene. The proband was an 11-year-old girl; examination revealed microcephaly, sloping nape, low hair growth on the forehead, flattened nose, wide columella, open mouth, adherent earlobes, breast enlargement, broad chest, small hands, narrow fingers, clinodactyly of the 5th fingers, valgus deformity of the feet, and flat feat. Exome sequencing identified a *de novo* missense substitution p.Tyr418Asp. The SpliceAI [44] predictor program predicted a possible gain of the splicing acceptor site that can lead to possible loss of function of RNA binding protein fox-1 homolog 1.

Patient D965. *SOX4* NM_003107.3:c.281G>A; NP_003098.1:p.(Gly94Asp)

De novo variants in the *SOX4* gene have been described in IDD with mild dysmorphism and clinodactyly of the 5th fingers. The authors noted a slight resemblance to mild Coffin-Siris syndrome [45]. The proband was a 5 year old girl. Examination revealed microcephaly, dolichocephaly, deep-set eyes, short filter, shortening and clinodactyly of the 5th fingers, left transverse palmar crease, and flat valgus feet. NGS identified a missense variant p.Gly94Asp was revealed. The variant was inherited from the mother with IDD and was not found in grandparents. It is located in the DNA-binding region and can disrupt the main function of transcription factor SOX-4.

Patient D965. *HUWE1* NM_031407.7:c.12719C>T; NP_113584.3:p.(Ser4240Phe)

Variants in the *HUWE1* gene cause an X-linked dominant disease, the clinical features of which are moderate to profound IDD, delayed or absent speech, short stature with short limbs, and facial dysmorphias (broad nasal tip, deep-set eyes, epicanthus, short palpebral fissures, and short filter) [46]. The patient was a 1.5-year-old boy with delayed motor and physical development. Examination revealed converging strabismus, epicanthus, narrow palpebral fissures, divergence of the rectus abdominis muscles, umbilical hernia, muscle hypotension, increased tendon reflexes

from the knees, uncertainty with holing objects in his hands, salivation. As a result of molecular genetic examination, a *de novo* missense variant p.Ser4240Phe was identified.

Patient D1059. *SMARCA4* NM_001128849.3:c.2933G>A; NP_001122321.1:p.(Arg978Gln)

In OMIM, the same MIM phenotype number 614609 corresponds to two disorders: autosomal dominant IDD type 16 and Coffin-Siris syndrome type 4. The patient was a 10-years-old boy with a history of delayed motor development and seizures. Examination revealed a flat nape, coarse hair, narrow face, fan-shaped thick eyebrows, thick eyelashes, closed fistulas in the inner corners of the eyes, rounded large tip of the nose, high palate, elbow joints with protruding ulnar heads, funnel chest deformity, absence of nail plates on the 5th toes. MRI revealed mixed hydrocephalus, hypogenesis of the corpus callosum, dysplastic cortical plate of the temporal lobes, retrocerebellar cyst. NGS identified a *de novo* missense variant p.Arg978Gln. This variant is not located in any functional region, but Aref-Eshghi et al. stated that the variant p.Arg978Gly at the same amino acid position had the same DNA methylation epi-signatures as other variants with the Coffin-Siris syndrome[47].

Patient D904. *KCNB1* in combination with a CNV NM_004975.4:c.1237G>A; NP_004966.1:p.(Val413Ile)

Pathogenic variants in the gene have been described in NIDD [48] and in early epileptic encephalopathy [49] without any phenotypic features. Our patient, a 9-year-old boy, had hypotonia and hypoplasia of the cerebellum. Examination revealed a narrow forehead, moderately upturned nose, macrostomia, low-set ears, transverse palmar crease, and varus feet. Exome sequencing identified a *de novo* mosaic missense variant p.Val413Ile. However, the phenotypic features of the patient do not correspond to the *KCNB1*-associated IDD, therefore, we decided to perform CMA, as a result of which we identified a heterozygous duplication 17p12 and deletion of 22q13.32q13.33 (arr[hg19]17p12(14087934_15484858)x3, 22q13.32q13.33(48571448_511978)x1) described in the Phelan McDermid syndrome, NIDD [50] and autism [51]. Thus, we were unable to classify the variant p.Val413Ile in the gene in any way, and therefore we did not describe it in the main text table with pathogenic and likely pathogenic variants.

Variants of unknown clinical significance which are the most likely cause of the disease.

Patient D381. *TRAPPC6B* NM_177452.4:c.119G>T; NP_803235.1:p.(Gly40Val)

Homozygous or compound heterozygous variants in the gene have been described in IDD with microcephaly, epilepsy, and cerebral atrophy. This is a rare autosomal recessive type of NIDD,

described in only 4 closely related families from Pakistan [52]. Our patients were siblings, a 4-year-old girl and a 6.5-year-old boy, born from a closely related marriage of natives of Tajikistan; they had microcephaly, flat occiput, delayed motor development and bouts of tonic tension in the arms during excitement. The girl underwent clinical exome sequencing, which did not identify any genetic variants. Whole exome sequencing identified a homozygous missense variant p.Gly40Val in the *TRAPPC6B*. Segregation in the family corresponded to the type of inheritance, however, the variant was classified as VUS, which is highly likely to be the cause of the disease. To prove its pathogenicity, it is necessary to conduct a functional analysis. The Human Splicing Finder predicts activation of a cryptic Donor splice site.Patient S4. *DYNC1H1* NM_001376.5:c.591_593del; NP_001367.2:p.(Gln198del)

Pathogenic variants in this gene are associated with autosomal dominant IDD, type 13, which can be accompanied by various clinical features, including microcephaly, brain anomalies, ataxic gait and seizures, however, these features have a very pronounced clinical heterogeneity [53]. We examined two women aged 45 and 65 (mother and daughter) with intellectual disabilities without any other clinical features. Sequencing of the daughter's exome identified the p.Q197del variant which was classified as VUS. This variant was also detected in the mother by the Sanger sequencing. The mother's parents were are not available for examination. This variant is located in the coiled coil structural motif of the cytoplasmic dynein 1 heavy chain 1.

Patient D954. *BCAP31* NM_001139441.1:c.716G>A; NP_001132913.1:p.(Gly239Asp)

This gene is located in Xq28 and encodes the BAP31 protein which plays an important role in anterograde transport from the endoplasmic reticulum to the Golgi apparatus. Patients with pathogenic variants in the gene have mild to severe IDD, dystonia, deafness, and central hypomyelination. Women are usually asymptomatic carriers, but in some cases they may have hearing loss [54]. The proband was a 13-years-old boy with a delayed motor development and hearing loss. Examination revealed microcephaly, thick eyebrows, protruding nose, high waist, hypoplasia of external genitalia, sandal-shaped fissure, hypoplasia of the 5th fingers were revealed. Exome sequencing revealed a hemizygous variant of unknown clinical significance p.Gly239Asp. The variant was also detected by Sanger sequencing in the boy's mother, aunt and maternal grandmother. The variant was not found in two healthy cousins of the mother, which did not allow us to confirm or exclude pathogenicity of this variant. Pathogenic variants in this gene are thought to lead to the loss of protein function, but there are also missense variants that are accompanied by milder clinical manifestations [54].

References

1. Cappuccio, G.; Sayou, C.; Tanno, P. Le; Tisserant, E.; Bruel, A.L.; Kennani, S. El; Sá, J.; Low, K.J.; Dias, C.; Havlovicová, M.; et al. De Novo SMARCA2 Variants Clustered Outside the Helicase Domain Cause a New Recognizable Syndrome with Intellectual Disability and Blepharophimosis Distinct from Nicolaides–Baraitser Syndrome. *Genet. Med.* **2020**, *22*, 1838–1850, doi:10.1038/s41436-020-0898-y.
2. Sousa, S.B.; Abdul-Rahman, O.A.; Bottani, A.; Cormier-Daire, V.; Fryer, A.; Gillessen-Kaesbach, G.; Horn, D.; Josifova, D.; Kuechler, A.; Lees, M.; et al. Nicolaides-Baraitser Syndrome: Delineation of the Phenotype. *Am. J. Med. Genet. Part A* **2009**, *149*, 1628–1640, doi:10.1002/ajmg.a.32956.
3. Santen, G.W.E.; Aten, E.; Vulto-van Silfhout, A.T.; Pottinger, C.; van Bon, B.W.M.; van Minderhout, I.J.H.M.; Snowdowne, R.; van der Lans, C.A.C.; Boogaard, M.; Linssen, M.M.L.; et al. Coffin-Siris Syndrome and the BAF Complex: Genotype-Phenotype Study in 63 Patients. *Hum. Mutat.* **2013**, *34*, 1519–1528, doi:10.1002/humu.22394.
4. Bell, S.; Rousseau, J.; Peng, H.; Aouabed, Z.; Priam, P.; Theroux, J.F.; Jefri, M.; Tanti, A.; Wu, H.; Kolobova, I.; et al. Mutations in ACTL6B Cause Neurodevelopmental Deficits and Epilepsy and Lead to Loss of Dendrites in Human Neurons. *Am. J. Hum. Genet.* **2019**, *104*, 815–834, doi:10.1016/j.ajhg.2019.03.022.
5. Fichera, M.; Failla, P.; Saccuzzo, L.; Miceli, M.; Salvo, E.; Castiglia, L.; Galesi, O.; Grillo, L.; Calì, F.; Greco, D.; et al. Mutations in ACTL6B, Coding for a Subunit of the Neuron-Specific Chromatin Remodeling Complex NBAF, Cause Early Onset Severe Developmental and Epileptic Encephalopathy with Brain Hypomyelination and Cerebellar Atrophy. *Hum. Genet.* **2019**, *138*, 187–198, doi:10.1007/s00439-019-01972-3.
6. Wenderski, W.; Wang, L.; Krokhotin, A.; Walsh, J.J.; Li, H.; Shoji, H.; Ghosh, S.; George, R.D.; Miller, E.L.; Elias, L.; et al. Loss of the Neural-Specific BAF Subunit ACTL6B Relieves Repression of Early Response Genes and Causes Recessive Autism. *Proc. Natl. Acad. Sci. U. S. A.* **2020**, *117*, 10055–10066, doi:10.1073/pnas.1908238117.
7. Bögershausen, N.; Wollnik, B. Mutational Landscapes and Phenotypic Spectrum of SWI/SNF-Related Intellectual Disability Disorders. *Front. Mol. Neurosci.* **2018**, *11*, 1–18, doi:10.3389/fnmol.2018.00252.
8. Seranski, P.; Heiss, N.S.; Dhorne-Pollet, S.; Radelof, U.; Korn, B.; Hennig, S.; Backes, E.; Schmidt, S.; Wiemann, S.; Schwarz, C.E.; et al. Transcription Mapping in a Medulloblastoma Breakpoint Interval and Smith-Magenis Syndrome Candidate Region: Identification of 53 Transcriptional Units and New Candidate Genes. *Genomics* **1999**, *56*, 1–11, doi:10.1006/geno.1998.5647.
9. Bi, W.; Saifi, G.M.; Shaw, C.J.; Walz, K.; Fonseca, P.; Wilson, M.; Potocki, L.; Lupski, J.R. Mutations of RAI1, a PHD-Containing Protein, in Nondeletion Patients with Smith-Magenis Syndrome. *Hum. Genet.* **2004**, *115*, 515–524, doi:10.1007/s00439-004-1187-6.
10. Gilissen, C.; Hehir-Kwa, J.Y.; Thung, D.T.; Van De Vorst, M.; Van Bon, B.W.M.; Willemsen, M.H.; Kwint, M.; Janssen, I.M.; Hoischen, A.; Schenck, A.; et al. Genome Sequencing Identifies Major Causes of Severe Intellectual Disability. *Nature* **2014**, *511*, 344–347, doi:10.1038/nature13394.
11. Brusco, A.; Bybjerg-grauholm, J.; Carracedo, A.; Chan, M.C.Y.; Chioc-chetti, A.G.; Chung, B.H.Y.; Cuccaro, M.L.; Curró, A.; Bernardina, B.D.; Doan, R.; et al. Large-Scale Exome Sequencing Study Implicates Both Developmental and Functional Changes in the Neurobiology of Autism. *Cell* **2021**, *180*, 568–584, doi:10.1016/j.cell.2019.12.036.Large-Scale.
12. Xu, Y.; Chen, W.; Amin, J.B.; Perozo, E.; Kannan, V.; Keller, S.R.; Wilcox, W.R.; Lemke, J.R.; Myers, J.; Swanger, S.A.; et al. De Novo GRIN Variants in NMDA Receptor M2 Channel Pore-Forming Loop Are Associated with Neurological Diseases. *Hum. Mutat.* **2020**, *40*, 2393–2413, doi:10.1002/humu.23895.De.

13. Lemke, J.R.; Geider, K.; Helbig, K.L.; Heyne, H.O.; Schütz, H.; Hentschel, J.; Courage, C.; Depienne, C.; Nava, C.; Heron, D.; et al. Delineating the GRIN1 Phenotypic Spectrum: A Distinct Genetic NMDA Receptor Encephalopathy. *Neurology* **2016**, *86*, 2171–2178, doi:10.1212/WNL.0000000000002740.
14. Stamberger, H.; Hammer, T.B.; Gardella, E.; Vlaskamp, D.R.M.; Bertelsen, B.; Mandelstam, S.; de Lange, I.; Zhang, J.; Myers, C.T.; Fenger, C.; et al. NEXMIF Encephalopathy: An X-Linked Disorder with Male and Female Phenotypic Patterns. *Genet. Med.* **2021**, *23*, 363–373, doi:10.1038/s41436-020-00988-9.
15. Magome, T.; Hattori, T.; Taniguchi, M.; Ishikawa, T.; Miyata, S.; Yamada, K.; Takamura, H.; Matsuzaki, S.; Ito, A.; Tohyama, M.; et al. XLMR Protein Related to Neurite Extension (Xpn/KIAA2022) Regulates Cell-Cell and Cell-Matrix Adhesion and Migration. *Neurochem. Int.* **2013**, *63*, 561–569, doi:10.1016/j.neuint.2013.09.011.
16. Bramswig, N.C.; Lüdecke, H.J.; Pettersson, M.; Albrecht, B.; Bernier, R.A.; Cremer, K.; Eichler, E.E.; Falkenstein, D.; Gerdts, J.; Jansen, S.; et al. Identification of New TRIP12 Variants and Detailed Clinical Evaluation of Individuals with Non-Syndromic Intellectual Disability with or without Autism. *Hum. Genet.* **2017**, *136*, 179–192, doi:10.1007/s00439-016-1743-x.
17. Jing Zhang, Tomasz Gambin, Bo Yuan, Przemyslaw Szafranski, Jill A. Rosenfeld, Mohammed Al Balwi, Abdulrahman Alswaid, Lihadh Al-Gazali, Aisha Al Shamsi, Makanko Komara, Bassam R. Ali, Elizabeth Roeder, Laura McAuley, Daniel S. Roy, David K. Manchester, Pi, and P.S. Haploinsufficiency of the E3 Ubiquitin-Protein Ligase Gene TRIP12 Causes Intellectual Disability with or without Autism Spectrum Disorders, Speech Delay, and Dysmorphic Features. *Physiol. Behav.* **2014**, *63*, 1–18, doi:10.1007/s00439-017-1763-1.Haploinsufficiency.
18. Knaus, A.; Awaya, T.; Helbig, I.; Afawi, Z.; Pendziwiat, M.; Abu-Rachma, J.; Thompson, M.D.; Cole, D.E.; Skinner, S.; Annese, F.; et al. Rare Noncoding Mutations Extend the Mutational Spectrum in the PGAP3 Subtype of Hyperphosphatasia with Mental Retardation Syndrome. *Hum. Mutat.* **2016**, *37*, 737–744, doi:10.1002/humu.23006.
19. Howard, M.F.; Murakami, Y.; Pagnamenta, A.T.; Daumer-Haas, C.; Fischer, B.; Hecht, J.; Keays, D.A.; Knight, S.J.L.; Kölsch, U.; Krüger, U.; et al. Mutations in PGAP3 Impair GPI-Anchor Maturation, Causing a Subtype of Hyperphosphatasia with Mental Retardation. *Am. J. Hum. Genet.* **2014**, *94*, 278–287, doi:10.1016/j.ajhg.2013.12.012.
20. Courcet, J.B.; Faivre, L.; Malzac, P.; Masurel-Paulet, A.; Lopez, E.; Callier, P.; Lambert, L.; Lemesle, M.; Thevenon, J.; Gigot, N.; et al. The DYRK1A Gene Is a Cause of Syndromic Intellectual Disability with Severe Microcephaly and Epilepsy. *J. Med. Genet.* **2012**, *49*, 731–736, doi:10.1136/jmedgenet-2012-101251.
21. Méjécase, C.; Way, C.M.; Owen, N.; Moosajee, M. Ocular Phenotype Associated with Dyrk1a Variants. *Genes (Basel).* **2021**, *12*, 1–14, doi:10.3390/genes12020234.
22. Gozes, I.; Van Dijck, A.; Hacohen-Kleiman, G.; Grigg, I.; Karmon, G.; Giladi, E.; Eger, M.; Gabet, Y.; Pasmanik-Chor, M.; Cappuyns, E.; et al. Premature Primary Tooth Eruption in Cognitive/Motor-Delayed ADNP-Mutated Children. *Transl. Psychiatry* **2017**, *7*, doi:10.1038/tp.2017.27.
23. Dijck, A. Van; Silfhout, A.T.V.; Cappuyns, E.; Werf, I.M. Van Der; Mancini, M.; Tzschach, A.; Bernier, R.; Gozes, I.; Eichler, E.E.; Lindstrand, A.; et al. Clinical Presentation of a Complex Neurodevelopmental Disorder Caused by Mutations in ADNP. *Biol Psychiatry* **2019**, *85*, 287–297, doi:10.1016/j.biopsych.2018.02.1173.Clinical.
24. Rentas, S.; Rathi, K.S.; Kaur, M.; Raman, P.; Krantz, I.D.; Sarmady, M.; Tayoun, A.A. Diagnosing Cornelia de Lange Syndrome and Related Neurodevelopmental Disorders Using RNA Sequencing. *Genet. Med.* **2020**, *22*, 927–936, doi:10.1038/s41436-019-0741-5.
25. Olley, G.; Ansari, M.; Bengani, H.; Grimes, G.R.; Rhodes, J.; von Kriegsheim, A.; Blatnik, A.; Stewart, F.J.; Wakeling, E.; Carroll, N.; et al. BRD4 Interacts with NIPBL and BRD4 Is Mutated in a Cornelia de Lange–like Syndrome. *Nat. Genet.* **2019**, *51*, 1192,

doi:10.1038/s41588-019-0448-1.
26. Jiang, Y.H.; Yuen, R.K.C.C.; Jin, X.; Wang, M.; Chen, N.; Wu, X.; Ju, J.; Mei, J.; Shi, Y.; He, M.; et al. Detection of Clinically Relevant Genetic Variants in Autism Spectrum Disorder by Whole-Genome Sequencing. *Am. J. Hum. Genet.* **2013**, *93*, 249–263, doi:10.1016/j.ajhg.2013.06.012.
27. Nakamura, K.; Kato, M.; Osaka, H.; Yamashita, S.; Nakagawa, E.; Haginoya, K.; Tohyama, J.; Okuda, M.; Wada, T.; Shimakawa, S.; et al. Clinical Spectrum of SCN2A Mutations Expanding to Ohtahara Syndrome. *Neurology* **2013**, *81*, 992–998, doi:10.1212/WNL.0b013e3182a43e57.
28. Rauch, A.; Wieczorek, D.; Graf, E.; Wieland, T.; Endele, S.; Schwarzmayr, T.; Albrecht, B.; Bartholdi, D.; Beygo, J.; Di Donato, N.; et al. Range of Genetic Mutations Associated with Severe Non-Syndromic Sporadic Intellectual Disability: An Exome Sequencing Study. *Lancet* **2012**, *380*, 1674–1682, doi:10.1016/S0140-6736(12)61480-9.
29. Reynolds, C.; King, M.D.; Gorman, K.M. The Phenotypic Spectrum of SCN2A-Related Epilepsy. *Eur. J. Paediatr. Neurol.* **2020**, *24*, 117–122, doi:10.1016/j.ejpn.2019.12.016.
30. Xia, F.; Bainbridge, M.N.; Tan, T.Y.; Wangler, M.F.; Scheuerle, A.E.; Zackai, E.H.; Harr, M.H.; Sutton, V.R.; Nalam, R.L.; Zhu, W.; et al. De Novo Truncating Mutations in AHDC1 in Individuals with Syndromic Expressive Language Delay, Hypotonia, and Sleep Apnea. *Am. J. Hum. Genet.* **2014**, *94*, 784–789, doi:10.1016/j.ajhg.2014.04.006.
31. Yang, H.; Douglas, G.; Monaghan, K.G.; Retterer, K.; Cho, M.T.; Escobar, L.F.; Tucker, M.E.; Stoler, J.; Rodan, L.H.; Stein, D.; et al. De Novo Truncating Variants in the AHDC1 Gene Encoding the AT-Hook DNA-Binding Motif-Containing Protein 1 Are Associated with Intellectual Disability and Developmental Delay. *Mol. Case Stud.* **2015**, *1*, a000562, doi:10.1101/mcs.a000562.
32. Tatton-Brown, K.; Zachariou, A.; Loveday, C.; Renwick, A.; Mahamdallie, S.; Aksglaede, L.; Baralle, D.; Barge-Schaapveld, D.; Blyth, M.; Bouma, M.; et al. The Tatton-Brown-Rahman Syndrome: A Clinical Study of 55 Individuals with de Novo Constitutive DNMT3A Variants. *Wellcome Open Res.* **2018**, *3*, 1–17, doi:10.12688/wellcomeopenres.14430.1.
33. White, J.; Beck, C.R.; Harel, T.; Posey, J.E.; Jhangiani, S.N.; Tang, S.; Farwell, K.D.; Powis, Z.; Mendelsohn, N.J.; Baker, J.A.; et al. POGZ Truncating Alleles Cause Syndromic Intellectual Disability. *Genome Med.* **2016**, *8*, 1–11, doi:10.1186/s13073-015-0253-0.
34. Stessman, H.A.F.; Willemsen, M.H.; Fenckova, M.; Penn, O.; Hoischen, A.; Xiong, B.; Wang, T.; Hoekzema, K.; Vives, L.; Vogel, I.; et al. Disruption of POGZ Is Associated with Intellectual Disability and Autism Spectrum Disorders. *Am. J. Hum. Genet.* **2016**, *98*, 541–552, doi:10.1016/j.ajhg.2016.02.004.
35. Zarate, Y.A.; Fish, J.L. SATB2-Associated Syndrome: Mechanisms, Phenotype, and Practical Recommendations. *Am. J. Med. Genet. Part A* **2017**, *173*, 327–337, doi:10.1002/ajmg.a.38022.
36. Farwell, K.D.; Shahmirzadi, L.; El-Khechen, D.; Powis, Z.; Chao, E.C.; Tippin Davis, B.; Baxter, R.M.; Zeng, W.; Mroske, C.; Parra, M.C.; et al. Enhanced Utility of Family-Centered Diagnostic Exome Sequencing with Inheritance Model-Based Analysis: Results from 500 Unselected Families with Undiagnosed Genetic Conditions. *Genet. Med.* **2015**, *17*, 578–586, doi:10.1038/gim.2014.154.
37. Lee, J.S.; Yoo, Y.; Lim, B.C.; Kim, K.J.; Choi, M.; Chae, J.H. SATB2-Associated Syndrome Presenting with Rett-like Phenotypes. *Clin. Genet.* **2016**, *89*, 728–732, doi:10.1111/cge.12698.
38. Breuss, M.; Heng, J.I.T.; Poirier, K.; Tian, G.; Jaglin, X.H.; Qu, Z.; Braun, A.; Gstrein, T.; Ngo, L.; Haas, M.; et al. Mutations in the β-Tubulin Gene TUBB5 Cause Microcephaly with Structural Brain Abnormalities. *Cell Rep.* **2012**, *2*, 1554–1562, doi:10.1016/j.celrep.2012.11.017.
39. Dentici, M.L.; Terracciano, A.; Bellacchio, E.; Capolino, R.; Novelli, A.; Digilio, M.C.;

Dallapiccola, B. Intrafamiliar Clinical Variability of Circumferential Skin Creases Kunze Type Caused by a Novel Heterozygous Mutation of N-Terminal TUBB Gene. *Clin. Genet.* **2018**, *93*, 1223–1228, doi:10.1111/cge.13232.
40. Cohen, J.S.; Srivastava, S.; Farwell Hagman, K.D.; Shinde, D.N.; Huether, R.; Darcy, D.; Wallerstein, R.; Houge, G.; Berland, S.; Monaghan, K.G.; et al. Further Evidence That de Novo Missense and Truncating Variants in ZBTB18 Cause Intellectual Disability with Variable Features. *Clin. Genet.* **2017**, *91*, 697–707, doi:10.1111/cge.12861.
41. Lopes, F.; Barbosa, M.; Ameur, A.; Soares, G.; De Sá, J.; Dias, A.I.; Oliveira, G.; Cabral, P.; Temudo, T.; Calado, E.; et al. Identification of Novel Genetic Causes of Rett Syndrome-like Phenotypes. *J. Med. Genet.* **2016**, *53*, 190–199, doi:10.1136/jmedgenet-2015-103568.
42. Piard, J.; Hu, J.H.; Campeau, P.M.; Rzońca, S.; Esch, H. Van; Vincent, E.; Han, M.; Rossignol, E.; Castaneda, J.; Chelly, J.; et al. FRMPD4 Mutations Cause X-Linked Intellectual Disability and Disrupt Dendritic Spine Morphogenesis. *Hum. Mol. Genet.* **2018**, *27*, 589–600, doi:10.1093/hmg/ddx426.
43. Kamien, B.; Lionel, A.C.; Bain, N.; Scherer, S.W.; Hunter, M. Outfoxed by RBFOX1-A Caution about Ascertainment Bias. *Am. J. Med. Genet. Part A* **2014**, *164*, 1411–1418, doi:10.1002/ajmg.a.36458.
44. Jaganathan, K.; Kyriazopoulou Panagiotopoulou, S.; McRae, J.F.; Darbandi, S.F.; Knowles, D.; Li, Y.I.; Kosmicki, J.A.; Arbelaez, J.; Cui, W.; Schwartz, G.B.; et al. Predicting Splicing from Primary Sequence with Deep Learning. *Cell* **2019**, *176*, 535-548.e24, doi:10.1016/j.cell.2018.12.015.
45. Zawerton, A.; Yao, B.; Yeager, J.P.; Pippucci, T.; Haseeb, A.; Smith, J.D.; Wischmann, L.; Kühl, S.J.; Dean, J.C.S.; Pilz, D.T.; et al. De Novo SOX4 Variants Cause a Neurodevelopmental Disease Associated with Mild Dysmorphism. *Am. J. Hum. Genet.* **2019**, *104*, 246–259, doi:10.1016/j.ajhg.2018.12.014.
46. Moortgat, S.; Berland, S.; Aukrust, I.; Maystadt, I.; Baker, L.; Benoit, V.; Caro-Llopis, A.; Cooper, N.S.; Debray, F.G.; Faivre, L.; et al. HUWE1 Variants Cause Dominant X-Linked Intellectual Disability: A Clinical Study of 21 Patients. *Eur. J. Hum. Genet.* **2018**, *26*, 64–74, doi:10.1038/s41431-017-0038-6.
47. Aref-Eshghi, E.; Bend, E.G.; Hood, R.L.; Schenkel, L.C.; Carere, D.A.; Chakrabarti, R.; Nagamani, S.C.S.; Cheung, S.W.; Campeau, P.M.; Prasad, C.; et al. BAFopathies' DNA Methylation Epi-Signatures Demonstrate Diagnostic Utility and Functional Continuum of Coffin–Siris and Nicolaides–Baraitser Syndromes. *Nat. Commun.* **2018**, *9*, doi:10.1038/s41467-018-07193-y.
48. Latypova, X.; Matsumoto, N.; Vinceslas-Muller, C.; Bézieau, S.; Isidor, B.; Miyake, N. Novel KCNB1 Mutation Associated with Non-Syndromic Intellectual Disability. *J. Hum. Genet.* **2017**, *62*, 569–573, doi:10.1038/jhg.2016.154.
49. Torkamani, A., Bersell, K., Jorge, B. S., Bjork, R. L., Jr, Friedman, J. R., Bloss, C. S., Cohen, J., Gupta, S., Naidu, S., Vanoye, C. G., George, A. L., Jr, Kearney, J.A. De Novo KCNB1 Mutations in Epileptic Encephalopathy. *Bone* **2008**, *23*, 1–7, doi:10.1002/ana.24263.De.
50. Gong, X.; Jiang, Y.W.; Zhang, X.; An, Y.; Zhang, J.; Wu, Y.; Wang, J.; Sun, Y.; Liu, Y.; Gao, X.; et al. High Proportion of 22q13 Deletions and SHANK3 Mutations in Chinese Patients with Intellectual Disability. *PLoS One* **2012**, *7*, doi:10.1371/journal.pone.0034739.
51. Chen, C.H.; Chen, H.I.; Liao, H.M.; Chen, Y.J.; Fang, J.S.; Lee, K.F.; Gau, S.S.F. Clinical and Molecular Characterization of Three Genomic Rearrangements at Chromosome 22q13.3 Associated with Autism Spectrum Disorder. *Psychiatr. Genet.* **2017**, *27*, 23–33, doi:10.1097/YPG.0000000000000151.
52. Marin-Valencia, I.; Novarino, G.; Johansen, A.; Rosti, B.; Issa, M.Y.; Musaev, D.; Bhat, G.; Scott, E.; Silhavy, J.L.; Stanley, V.; et al. A Homozygous Founder Mutation in TRAPPC6B Associates with a Neurodevelopmental Disorder Characterised by Microcephaly, Epilepsy and Autistic Features. *J. Med. Genet.* **2018**, *55*, 48–54, doi:10.1136/jmedgenet-2017-104627.

53. Anazi, S.; Maddirevula, S.; Salpietro, V.; Asi, Y.T.; Alsahli, S.; Alhashem, A. Expanding the Genetic Heterogeneity of Intellectual Disability. *Hum. Genet.* **2017**, *136*, 1419–1429, doi:10.1007/s00439-017-1843-2.
54. Whalen, S.; Shaw, M.; Mignot, C.; Héron, D.; Bastaraud, S.C.; Walti, C.C.; Liebelt, J.; Elmslie, F.; Yap, P.; Hurst, J.; et al. Further Delineation of BCAP31-Linked Intellectual Disability: Description of 17 New Families with LoF and Missense Variants. *Eur. J. Hum. Genet.* **2021**, *29*, 1405–1417, doi:10.1038/s41431-021-00821-0.
